# Fœtus, with Left Arm Partly Amputated by the Funis Coiled Round It

**Published:** 1847-04

**Authors:** 


*Fœtus, with left Arm partly amputated by the Funis coiled round it.* (Dub. Quart. Jour. of Med. Scien., Nov., 1846.)—Dr. Beatty exhibited to the Pathological Society of Dublin, a preparation of a fœtus, of between the fifth and sixth months of utero-gestation, which he adduced in confirmation of the fact several years ago described by Dr. Montgomery, the spontaneous amputation of the limbs before birth. This specimen illustrates that process, and may help to explain why the fœtus is so often found maimed before it arrives at its full period. The left arm in this instance had been enveloped in a coil of the umbilical cord, which had so tightly constricted it as to cause the absorption of all the tissues with which it was in contact; this had occurred about the middle of the humerus, and, the soft part being removed, only the bone preserved the continuity of the limb.—*Ibid.*

